# Type I feline coronavirus spike glycoprotein fails to recognize aminopeptidase N as a functional receptor on feline cell lines

**DOI:** 10.1099/vir.0.82666-0

**Published:** 2007-06

**Authors:** Charlotte Dye, Nigel Temperton, Stuart G. Siddell

**Affiliations:** 1 Division of Virology, Department of Cellular and Molecular Medicine, School of Medical and Veterinary Sciences, University of Bristol, Bristol BS8 1TD, UK; 2 MRC/UCL Centre for Medical Molecular Virology, University College London, 46 Cleveland Street, London W1T 4JF, UK

## Abstract

There are two types of feline coronaviruses that can be distinguished by serology and sequence analysis. Type I viruses, which are prevalent in the field but are difficult to isolate and propagate in cell culture, and type II viruses, which are less prevalent but replicate well in cell culture. An important determinant of coronavirus infection, *in vivo* and in cell culture, is the interaction of the virus surface glycoprotein with a cellular receptor. It is generally accepted that feline aminopeptidase N can act as a receptor for the attachment and entry of type II strains, and it has been proposed that the same molecule acts as a receptor for type I viruses. However, the experimental data are inconclusive. The aim of the studies reported here was to provide evidence for or against the involvement of feline aminopeptidase N as a receptor for type I feline coronaviruses. Our approach was to produce retroviral pseudotypes that bear the type I or type II feline coronavirus surface glycoprotein and to screen a range of feline cell lines for the expression of a functional receptor for attachment and entry. Our results show that type I feline coronavirus surface glycoprotein fails to recognize feline aminopeptidase N as a functional receptor on three continuous feline cell lines. This suggests that feline aminopeptidase N is not a receptor for type I feline coronaviruses. Our results also indicate that it should be possible to use retroviral pseudotypes to identify and characterize the cellular receptor for type I feline coronaviruses.

## INTRODUCTION

Coronaviruses are enveloped, plus-stranded RNA viruses that cause widespread disease in humans and animals. They tend to infect just one or a few closely related species and in their natural host exhibit marked tissue tropism. Often, the respiratory or gut epithelia are specifically targeted, causing localized infection, but disseminated infections leading to systemic disease are also seen. Together with toroviruses, arteriviruses and roniviruses, coronaviruses are members of the order *Nidovirales* (Siddell *et al.*, 2005).

Feline coronavirus (FCoV) infection is extremely common in cats. Natural infections with FCoV are usually transient, although a significant percentage of infections may become persistent (Addie & Jarrett, 2001). Most infections are asymptomatic or result in mild, self-limiting gastrointestinal disease, and in these cases the causative agent is known as feline enteric coronavirus (FECV). In a small percentage (<5 %) of animals, however, a fatal multi-systemic, immune-mediated disease occurs and this is known as feline infectious peritonitis (FIP) (Pedersen, 1995). Virus associated with FIP is referred to as feline infectious peritonitis virus (FIPV).

There are two types of FCoV that can be distinguished by serology and sequence analysis. Type I viruses are most prevalent in the field and account for approximately 80 % of all infections (Addie *et al.*, 2003; Hohdatsu *et al.*, 1992). Type II viruses are less prevalent and are characterized by recombination events that result in the replacement of the FCoV spike (S) glycoprotein gene with the equivalent gene of canine enteric coronavirus (CCoV) (Herrewegh *et al.*, 1998). There is no evidence that either type is more commonly associated with FIP in natural infections (Benetka *et al.*, 2004). The majority of research on FCoV to date has concentrated on the investigation of type II strains, most notably FIPV 79-1146, because they replicate well in cell culture.

The species specificity of coronaviruses is, to a large extent, determined by the recognition of a functional receptor on the surface of the host cell (Kuo *et al.*, 2000). Coronaviruses use a variety of cellular receptors (Masters, 2006) and, even within a single host species, different receptors are used by different coronaviruses (Li *et al.*, 2003; Yeager *et al.*, 1992). It is accepted that type II FCoV strains use feline aminopeptidase N (fAPN) as a receptor for host attachment and entry (Tresnan *et al.*, 1996). Consequently, they can be readily propagated in cell lines such as Crandell feline kidney (CrFK) cells, which express fAPN on their surface (Miguel *et al.*, 2002). In contrast, there is conflicting evidence regarding the receptor for the attachment and entry of type I FCoV. Tresnan *et al.* (1996) have reported that the UCD-1 strain of FIPV, which is a type I virus, also uses fAPN as a receptor, albeit inefficiently. Their conclusion was based upon the ability of the UCD-1 strain to infect and express viral antigens in both hamster and mouse cells (which, normally, cannot be infected) that had been stably transfected with fAPN cDNA. In contrast, Hohdatsu *et al.* (1998) have concluded that fAPN is not a receptor for type I FCoV. Their conclusion is based upon the ability of an fAPN-specific monoclonal antibody (mAb), R-G-4, to block the infection of *Felis catus* whole foetus (Fcwf-4) cells with type II viruses, whereas the same antibody was not able to block infection with type I viruses.

The aim of the studies reported here was to provide evidence for or against the involvement of fAPN as a receptor for type I FCoV. Our approach was to produce retroviral pseudotypes that bear type I or type II FCoV S glycoprotein and produce a green fluorescent protein (GFP) reporter gene signal in transduced cells. We chose this approach because human coronavirus S glycoproteins have been successfully pseudotyped onto similar retroviral vectors and then used to analyse the recognition of cellular receptors (Hofmann *et al.*, 2006; Simmons *et al.*, 2004; Temperton *et al.*, 2005). In our case, the pseudotypes were used to screen a range of feline cell lines for the expression of a functional receptor for attachment and entry. Our results clearly show that type I FCoV S glycoprotein fails to recognize fAPN as a functional receptor on three continuous feline cell lines. This suggests fAPN is not the receptor for type I FCoV. Our results also demonstrate that these retroviral pseudotypes can be used to screen for cells that are permissive for attachment and entry with FCoV, and we conclude that they can be used to identify and characterize the cellular receptor for type I FCoV. This would allow for the development of cell lines that efficiently replicate and propagate type I FCoV, which, in turn, would facilitate the investigation of these more clinically relevant viruses and aid in the development of a type I FCoV reverse genetics system.

## METHODS

### Cell lines and antibodies.

Cultured cell lines were maintained in Dulbecco’s modified Eagle’s medium with 10–15 % fetal bovine serum (FBS), 100 U penicillin G sulphate ml^−1^, 100 μg streptomycin sulphate ml^−1^ and non-essential amino acids. For routine growth, cell monolayers in 75 cm^2^ tissue culture flasks were kept at 37 °C in 5 % CO_2_ humidified incubators. All cell lines were adherent and were passaged when confluent. CrFK, Fcwf-4, swine testicular (ST) and human embryonic kidney (HEK 293T) cells were purchased from the ATCC. Feline kidney Colorado University (FKCU) cells were a gift from T. Gruffydd-Jones (Division of Companion Animal Studies, University of Bristol, Langford House, Langford, BS40 5DU, UK). The FCoV type II S glycoprotein-specific mAb 23F4.4 (Corapi *et al.*, 1992) was a gift from P. Rottier (Virology Division, Department of Infectious Diseases and Immunology, Yalelaan 1, 3584 CL Utrecht, The Netherlands). The FCoV type I hyperimmune polyclonal serum 210-70-FIP1 was purchased from VMRD. AlexaFluor-488 goat anti-mouse IgG and fluorescein isothiocyanate (FITC)-conjugated goat anti-cat IgG were purchased from Invitrogen and Jackson ImmunoResearch, respectively. Alkaline phosphatase (AP)-conjugated goat anti-cat IgG was also purchased from Jackson ImmunoResearch.

### Isolation of viral RNA.

The type II laboratory strain, FIPV 79-1146 (P100), was purchased from ATCC and virus stocks were prepared by infecting subconfluent CrFK cell monolayers at a high m.o.i. Cell culture supernatant containing virus was harvested at 8 h post-infection, centrifuged at 800 ***g*** for 5 min and stored at −80 °C. RNA was isolated from the cell culture supernatant using a QIAamp viral RNA mini kit (Qiagen) according to the manufacturer’s instructions and the eluted RNA was stored at −80 °C. Type I FCoV RNA was extracted at post-mortem from the jejunum of a cat with a histopathologically confirmed diagnosis of FIP. A 0.5 cm^3^ cube of jejunum was placed into 2 ml RNAlater solution (Qiagen) and stored at 4 °C overnight. The RNAlater solution was then discarded and the tissue stored at −80 °C. RNA was isolated from the tissue biopsy using a Macherey-Nagel Nucleospin RNA II extraction kit (Macherey-Nagel) according to the manufacturer’s instructions. RNA was stored at −80 °C.

### Amplification of viral RNA.

Primers for PCR amplification of the FCoV S genes were designed using the Lasergene-6 Primer Design software (dnastar). In order to facilitate cloning of PCR products into the pCAGGS expression vector (Niwa *et al.*, 1991), the forward and reverse primers were designed to incorporate *Kpn*I and *Xho*I restriction sites, respectively. The forward primers were also designed with a preferred Kozak sequence (GCCACCAUG) (Kozak, 1987) incorporating the S gene start codon (see Supplementary Table S1, available in JGV Online). The FCoV S genes were amplified using a Superscript One-Step RT-PCR for Long Templates kit (Invitrogen) according to the manufacturer’s instructions. Briefly, a 50 μl reaction containing RNA, 15 pmol forward primer, 15 pmol reverse primer, 1 μl Superscript II RT/Platinum *Taq* HiFi DNA polymerase enzyme mix and 1× reaction buffer was incubated at 50 °C for 30 min and 94 °C for 2 min, followed by 35 cycles of 94 °C for 15 s, 55 °C for 30 s and 68 °C for 4.75 min. The reaction was held at 72 °C for 7 min and then stored at 4 °C. Primer removal was done using a Qiagen PCR Purification kit according to the manufacturer’s instructions.

### Recombinant DNA and sequencing.

Recombinant DNA procedures, including gel electrophoresis, restriction enzyme digestion, modification and ligation of DNA, transformation of chemically competent cells and plasmid DNA isolation followed standard procedures (Ausubel *et al.*, 1987) or were done according to the manufacturer’s instructions. PCR products were cloned into pCR-Blunt II-TOPO vector DNA (Invitrogen) and DNA fragments were extracted from agarose gel using a Qiagen gel purification kit. Thermal cycle sequencing of PCR products and plasmid DNA was done with ABI v3.1 BIG DYE mix (Applied Biosystems) and, following primer removal, the reaction products were analysed with an ABI 310 Genetic Analyzer. Sequencing primers were designed from the FIPV 79-1146 and the FCoV C1Je S glycoprotein gene sequences; GenBank accession nos DQ010921 and DQ848678 (Dye & Siddell, 2005), and published vector sequences (see Supplementary Table S1, available in JGV Online). Computer-assisted analysis of sequence data was done using the Lasergene-6 Seqman software (dnastar).

### Transfection of HEK 293T cells with plasmid DNA.

HEK 293T cells were grown to 50–80 % confluency in six-well culture plates or six-well culture plates containing glass coverslips. Fugene-6 transfection reagent (Roche) was diluted (3 : 100) in OptiMEM (Invitrogen), mixed and incubated at room temperature for 5 min. Following the addition of plasmid DNA (1 μg per 100 μl diluted Fugene-6 reagent), the tube was mixed again and incubated at room temperature for a further 30 min. The Fugene-6/OptiMEM/DNA mixture (100 μl) was added to 5×10^5^ cells in a drop-wise fashion and the plates were agitated gently to ensure homogeneous mixing. Cells were then incubated for 48 h at 37 °C.

### Immunofluorescence for the detection of viral S glycoproteins.

Transfected cells were washed twice with cold PBS and incubated at room temperature for 15 min with PBS containing 4 % methanol-stabilized formaldehyde. Cells were then washed three times with PBS and incubated at 4 °C for 1 h with saponin buffer (PBS containing 0.1 % saponin, 0.1 % sodium azide and 10 % FBS). The fixed, permeablized cells were incubated at 4 °C for 1 h with primary antibody (diluted 1 : 100 in saponin buffer), washed three times with saponin buffer and then incubated at room temperature for 30 min with a secondary antibody (diluted 1 : 10 000 in saponin buffer). After washing three times in saponin buffer, the coverslips were mounted in Vectasheild mounting medium with 4,6-diamidino-2-phenylindole (DAPI; Vector) and viewed under a fluorescence microscope.

### Fluorescence-activated cell sorting (FACS) analysis for the detection of cell surface expressed viral S glycoproteins.

Transfected cells were trypsinized, resuspended in medium and centrifuged at 400 ***g*** for 5 min at room temperature. The cell pellet was washed twice in staining buffer (PBS containing 0.1 % sodium azide and 2 % FBS) and the cells were resuspended in primary antibody (diluted 1 : 100 in staining buffer) at 8×10^6^ cells ml^−1^. After a 30 min incubation at 4 °C, the cells were washed with staining buffer and resuspended in secondary antibody (diluted 1 : 10 000 in staining buffer) at 8×10^6^ cells ml^−1^. Cells were incubated in the dark at 4 °C for 20 min and then washed with staining buffer. Cells were resuspended in staining buffer at 8×10^5^ cells ml^−1^ and propidium iodide (PI) viability stain was added to a concentration of 50 μg ml^−1^. Cell suspensions were placed on ice in the dark and analysed by flow cytometry using the FACScan system. Non-viable PI-positive cells were excluded based on light scatter and the remaining gated cells were analysed for fluorescence. Data were acquired and analysed using the Cell Quest software (BD Biosciences).

### Production of viral pseudotypes.

Retroviral pseudotypes were produced using the three plasmid, transient transfection method in which HEK 293T cells were co-transfected with three plasmids expressing the murine leukemia virus (MLV) *gag*/*pol* genes (pCMVi), the FCoV S glycoprotein (this paper) or vesicular stomatitis virus-G protein genes (pMDG) and an enhanced GFP reporter construct (pCNCG), respectively (Naldini *et al.*, 1996; Towers *et al.*, 2000). Briefly, HEK 293T cells were grown to 80 % confluency in 25 cm^2^ dishes. A mixture of the three plasmid DNA constructs (see Supplementary Table S2, available in JGV Online) was prepared and added to a Fugene-6/OptiMEM mixture. The Fugene-6/OptiMEM/DNA mixture was incubated at room temperature and was added to the cells in a drop-wise fashion. Plates were agitated gently to ensure homogeneous mixing and the cells were then incubated at 37 °C in 5 % CO_2_. Supernatant was harvested 48 and 72 h post-transfection, filtered through 0.45 μm filters and stored at −80 °C.

### Transduction of cultured cell lines with viral pseudotypes.

Cell culture supernatant containing viral pseudotypes was supplemented with 160 μg Polybrene (Chemicon) ml^−1^ and added to subconfluent (50–80 %) cell monolayers [50 μl (ml cell culture medium)^−1^] in a drop-wise fashion. Plates were agitated gently to ensure homogeneous mixing and the cells were incubated for 72 h at 37 °C in 5 % CO_2_. The expression of GFP was monitored at 520 nm with a Nikon Eclipse TS100 microscope.

### ELISA for the detection of S glycoprotein on viral pseudotypes.

ELISA microtitre plates were coated overnight at 4 °C with cell culture supernatant containing retroviral pseudotypes diluted in coating buffer (100 mM Na_2_CO_3_, 100 mM NaHCO_3_, pH 9.6). Plates were washed with PBS containing 0.1 % Tween 20 and residual protein binding was blocked with blocking solution (PBS containing 0.1 % Tween 20, 1 % BSA) for 1 h at 21 °C. Duplicate twofold dilutions of primary antibody in blocking solution (1 : 10–1 : 2560) were added to antigen-coated and uncoated wells at 21 °C for 1.5 h. Plates were washed three times with PBS containing 0.1 % Tween 20 and AP-conjugated secondary antibody diluted 1 : 10 000 in PBS containing 0.05 % Tween 20 was added. After 1 h at 21 °C, plates were washed three times with PBS containing 0.1 % Tween 20. ELISAs were developed with Sigma Fast pNPP tablets (1.0 mg pNPP ml^−1^, 0.2 M Tris/HCl, pH 8.0 buffer). Following a 1 h incubation at 37 °C, plates were read at 405 nm.

### Transduction of feline adherent polymorphonuclear cell cultures with viral pseudotypes.

Heparinized whole blood was taken from specific-pathogen-free cats and 15 ml aliquots were mixed with an equal volume of RPMI 1640 medium and layered on to 10 ml Ficoll-Paque PLUS (GE Healthcare). After centrifugation for 40 min at 400 ***g***, the upper serum layer was removed, the polymorphonuclear cell layer was harvested and then washed four times in RPMI 1640 medium. Cells were counted and taken up in RPMI 1640 medium containing 10 % FBS, 1000 U penicillin ml^−1^ and 1000 μg streptomycin ml^−1^ to give a cell concentration of 2×10^6^ cells ml^−1^. Aliquots of 250 μl were added to wells of a 24-well culture plate. After 24 h, the medium was removed and replaced with 0.5 ml fresh medium. This process was repeated daily for 3 days and then twice weekly.

Cell culture supernatant containing viral pseudotypes was supplemented with 160 μg Polybrene (Chemicon) ml^−1^ and added [50 μl (ml cell culture medium)^−1^] in a drop-wise fashion to adherent feline polymorphonuclear cell cultures that had been maintained for 7–14 days. Plates were agitated gently to ensure homogeneous mixing and the cells were incubated for 72 h at 37 °C in 5 % CO_2_. The expression of GFP was monitored at 520 nm with a Nikon Eclipse TS100 microscope.

## RESULTS

### Amplification and cloning of the FCoV types I and II S genes

RNA was isolated from FIPV 79-1146-infected cell culture supernatant and from the jejunum of a cat with FIP. The type II and type I FCoV S genes were amplified respectively in one-step RT-PCR reactions. Primer sets T003/T004 and T013/T014 were used to produce truncated cDNA versions of the type II (4347 bp) and type I (4392 bp) S genes. The truncated versions of the S glycoprotein genes terminated 11 codons upstream of the authentic S gene stop codon and eliminated a dibasic retention signal (KXHXX, KVHV/IH), which is proposed to contribute to the localization of FCoV S glycoprotein to the site of virus assembly at the endoplasmic reticulum–Golgi intermediate compartment (Lontok *et al.*, 2004). Primer sets T009/T010 and T013/T015 were used to produce full-length versions of the type II (4383 bp) and type I (4428 bp) S genes. Each of the four S gene PCR cDNA fragments was then cloned into the Blunt II-TOPO vector and colonies containing the correct plasmid inserts were identified by restriction enzyme analysis. Sequencing the S gene cDNA inserts was done using FCoV type II and type I S gene-specific primers and the Blunt II-TOPO vector-specific primers M13-F and M13-R. This revealed the presence of mutations in each construct. Before proceeding, these mutations were repaired by the exchange of restriction fragments from cDNA clones with authentic S glycoprotein gene sequences. Because of the position of the mutations and a lack of convenient restriction sites in the type I S gene constructs, silent mutations at positions 474 (A→G) and 1827 (T→C) were not repaired. Plasmid DNA from the four Blunt II-TOPO S gene constructs was digested using *Kpn*I and *Xho*I and the S gene fragments were ligated into the multiple cloning site of pCAGGS vector DNA. The correct plasmid inserts were identified by restriction enzyme analysis, and nucleotide sequencing confirmed that all four inserts had the expected S gene nucleotide sequence. The vector–cDNA junctions were also verified by sequencing with vector-specific primers, pCAGG-F and pCAGG-R (see Supplementary Table S1, available in JGV Online). The constructs were designated pCAGGS/FCoVII-Str (type II, truncated), pCAGGS/FCoVII-S (type II, full-length), pCAGGS/FCoVI-Str (type I, truncated) and pCAGGS/FCoVI-S (type I, full-length).

### Intracellular FCoV S gene expression using immunofluorescence assay (IFA)

**Fig. 1. f1:**
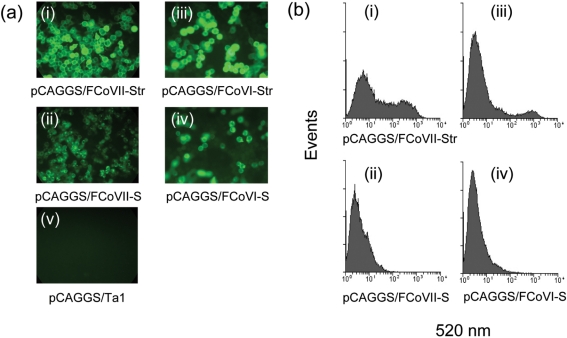
Intracellular and cell surface expression of FCoV surface glycoproteins. (a) HEK 293T cells grown on coverslips were transfected with (i) pCAGGS/FCoVII-Str, (ii) pCAGGS/FCoVII-S, (iii) pCAGGS/FCoVI-Str, (iv) pCAGGS/FCoVI-S or (v) pCAGGS/Ta1 plasmid DNA. Cells were permeabilized and stained 48 h post-transfection using 23F4.4 mAb [(i), (ii) and (v)] or 210-70-FIP1 polyclonal serum [(iii) and (iv)] as the primary antibody and AlexaFluor-488 goat anti-mouse IgG [(i), (ii) and (v)] or FITC-conjugated goat anti-cat IgG [(iii) and (iv)] as the secondary antibody. Stained cells were viewed using a fluorescence microscope. (b) HEK 293T cells were transfected with (i) pCAGGS/FCoVII-Str, (ii) pCAGGS/FCoVII-S, (iii) pCAGGS/FCoVI-Str or (iv) pCAGGS/FCoVI-S, or plasmid DNA. Cells were trypsinized and stained 48 h post-transfection using the 23F4.4 mAb [(i) and (ii)] or the 210-70-FIP1 polyclonal serum [(iii) and (iv)] as the primary antibody and AlexaFluor-488 goat anti-mouse IgG [(i) and (ii)] or FITC-conjugated goat anti-cat IgG [(iii) and (iv)] as the secondary antibody. Cells were analysed for fluorescence by flow cytometry using the FACScan system.

IFA was used to confirm FCoV S gene expression following the transfection of HEK 239T cells with the pCAGGS expression plasmids. HEK 293T cells grown to 80 % confluency on coverslips were transfected with pCAGGS/FCoVII-Str, pCAGGS/FCoVII-S, pCAGGS/FCoVI-Str and pCAGGS/FCoVI-S plasmid DNA, or with pCAGGS/Ta1 plasmid DNA (N. Temperton, unpublished) as a negative control. IFA was done 48 h post-transfection using the 23F4.4 mAb or the 210-70-FIP1 polyclonal serum as the primary antibody and AlexaFluor-488 goat anti-mouse IgG or FITC-conjugated goat anti-cat IgG as the secondary antibody. When viewed under the fluorescence microscope, a strong fluorescence signal was present in approximately 30–40 % of the pCAGGS/FCoVII-Str and pCAGGS/FCoVII-S transfected cells, and approximately 20–25 % of the pCAGGS/FCoVI-Str and pCAGGS/FCoVI-S transfected cells. No fluorescence was evident in the pCAGGS/Ta1 transfected negative-control cells (Fig. 1a[Fig f1]).

### Cell surface FCoV S gene expression

During the formation of retroviral pseudotypes, the surface protein from a heterologous virus is likely to be incorporated into the viral particle, predominantly during budding of the virus from the cell surface. For surface proteins to be effectively incorporated into the viral pseudotype, it is therefore preferable that they are expressed at the host cell membrane. HEK 293T cells were transfected with pCAGGS/FCoVII-Str, pCAGGS/FCoVII-S, pCAGGS/FCoVI-Str and pCAGGS/FCoVI-S plasmid DNA, and FACS analysis was used to quantify surface expression of the FCoV S glycoproteins 48 h post-transfection. The results show that the highest levels of S glycoprotein surface expression were obtained in the cells transfected with the truncated S gene constructs. Approximately, 41 % of cells transfected with the truncated type II S gene plasmid (pCAGGS/FCoVII-Str) were fluorescence positive, in contrast to only 2 % of cells transfected with the full-length type II S gene plasmid (pCAGGS/FCoVII-S). Similarly, 11 % of cells transfected with the truncated type I S gene plasmid (pCAGGS/FCoVI-Str) were fluorescence positive, in contrast to only 3 % of cells transfected with the full-length version (pCAGGS/FCoVI-S) (Fig. 1b[Fig f1]). These results demonstrate that truncated forms of the FCoV S glycoprotein should be used to produce high titre viral pseudotypes and they support the idea that the dibasic motif identified at the carboxyl terminus of the FCoV S glycoprotein (Lontok *et al.*, 2004) is indeed an intracellular retention signal.

### Cellular tropism of viral pseudotypes

**Fig. 2. f2:**
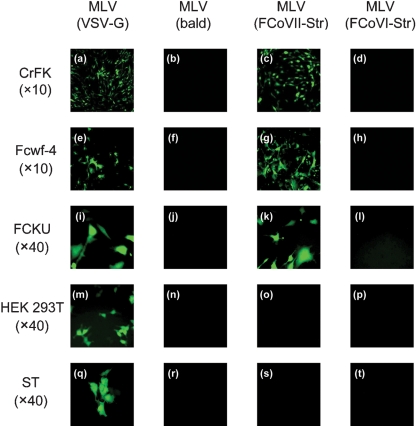
Cellular tropism of viral pseudotypes. CrFK (a, b, c and d), Fcwf-4 (e, f, g and h), FCKU (i, j, k and l), HEK 293T (m, n, o and p) and ST (q, r, s and t) cells were incubated with MLV(VSV-G) (a, e, i m and q), MLV(bald) (b, f, j, n and r), MLV(FCoVII-Str) (c, g, k, o and s) or MLV(FCoVI-Str) (d, h, l, p and t) viral pseudotypes. At 72 h post-incubation, the cells were analysed for GFP expression at 520 nm using a ×10 (a to h) or ×40 (i to t) objective on a Nikon Eclipse TS100 fluorescence microscope.

Four MLV-based viral pseudotypes were produced: pseudotypes containing the truncated forms of either the FCoV type I or type II S glycoproteins [MLV(FCoVI-Str) and MLV(FCoVII-Str)], pseudotype containing the VSV-G surface protein [MLV(VSV-G), positive control] and psuedotype with no surface protein, which we refer to as ‘bald’ pseudotype [MLV(bald)]. Three established feline continuous cell lines (CrFK, Fcwf-4 and FKCU), ST and HEK 293T cells were grown to 80 % confluency and incubated with each of the viral pseudotypes. At 72 h post-transduction, the cells were monitored for GFP expression. In all cell lines, GFP expression could be detected in cells transduced with the MLV(VSV-G) viral pseudotype. Also as expected, no GFP expression could be detected in cells incubated with MLV(bald) pseudotype. The FCoV type II S pseudotype, MLV(FCoVII-Str), was able to transduce GFP expression in CrFK, Fcwf-4 and FKCU cells but not in ST or HEK 293T cells. And finally, there was no evidence of GFP expression in any of the cell lines that had been incubated with the FCoV type I S pseudotype, MLV(FCoVI-Str) (Fig. 2).

**Fig. 3. f3:**
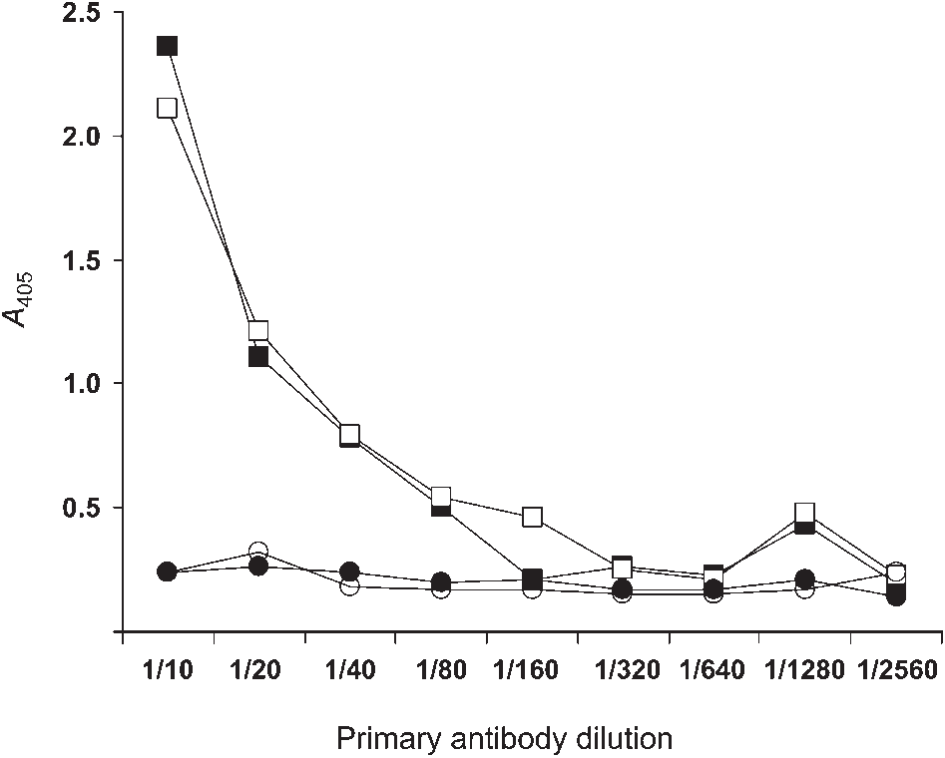
ELISA of FCoV type I S glycoprotein in MLV(FCoVI-Str) retroviral pseudotypes. ELISA microtitre plates were coated with cell culture supernatant containing a 1 : 32 dilution MLV(FCoVI-Str) retroviral pseudotypes diluted in coating buffer (▪), a 1 : 64 dilution MLV(FCoVI-Str) retroviral pseudotypes diluted in coating buffer (□), cell culture supernatant diluted in coating buffer (•) or coating buffer alone (○). Duplicate twofold dilutions of primary antibody (1 : 10–1 : 2560) were incubated with antigen-coated and uncoated wells and bound antibody was detected with AP-conjugated secondary antibody and pNPP. Plates were read at 405 nm.

These results suggest to us that the feline cell lines tested did not express a receptor for the type I FCoV S glycoprotein. In contrast, they were clearly transduced with pseudotypes bearing the FCoV type II S glycoprotein, which is known to use fAPN as a receptor for attachment and entry. However, as the levels of S glycoprotein expression in HEK 239T cells transfected with both type I and type II truncated S glycoprotein constructs were not the same (Fig. 1), it is possible that, compared with the FCoV type II pseudotypes, the FCoV type I pseudotypes had failed to incorporate a sufficient density of membrane-bound S glycoprotein. To address this, two further experiments were done. First, an ELISA assay was done to detect the presence of the FCoV type I S glycoprotein in the FCoV type I pseudotype particles. ELISA plates were coated with the MLV(FCoVI-Str) pseudotype supernatant diluted in coating buffer and the 210-70-FIP1 antiserum was used as the primary antibody. The results are shown in Fig. 3. The two control samples (coating buffer only and coating with medium) were negative over the range of dilutions tested. In contrast, at two different antigen concentrations, the FCoV type I pseudotype supernatant showed a positive signal relative to the primary antibody concentration. These results indicate that type I S glycoprotein is present within the FCoV type I pseudotype supernatant, and most likely associated with retroviral cores.

**Fig. 4. f4:**
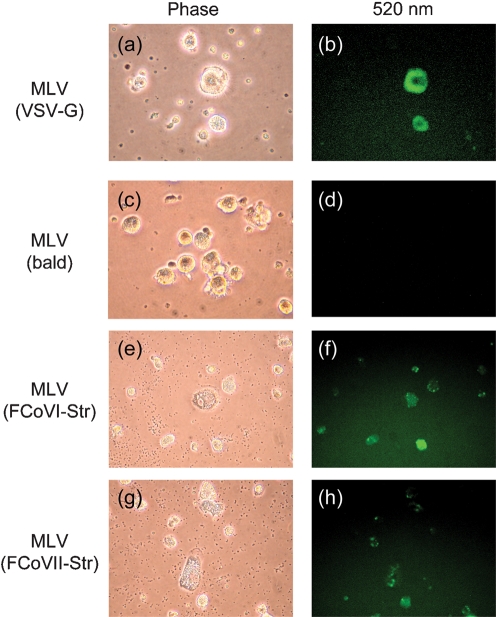
Transduction of feline adherent polymorphonuclear cells with MLV(FCoVI-Str) retroviral pseudotypes. Feline adherent polymorphonuclear cell cultures were incubated with MLV(VSV-G) (a and b), MLV(bald) (c and d), MLV(FCoVI-Str) (e and f) or MLV(FCoVII-Str) (g and h) viral pseudotypes. At 72 h post-incubation, the cells were visualized by microscopy (a, c, e and g) and analysed for GFP expression at 520 nm (b, d, f and h) using a Nikon Eclipse TS100 fluorescence microscope.

Second, and more importantly, the retroviral pseudotypes were used to transduce cultures of adherent polymorphonuclear cells that had been obtained from the blood of specific-pathogen-free cats. These primary cell cultures were cultivated without growth factors (e.g. interleukin-4 and granulocyte–macrophage colony-stimulating factor) and were mainly of monocyte morphology. The results are shown in Fig. 4. In these cultures, GFP expression was detected in cells transduced with the MLV(VSV-G) viral pseudotype and, as expected, no GFP expression could be detected in cells incubated with MLV(bald) pseudotypes. Both the FCoV type I S pseudotype, MLV(FCoVI-Str) and the FCoV type II S pseudotype, MLV(FCoVII-Str), were able to transduce GFP expression in a significant proportion of cells. This shows that the FCoV type I S pseudotypes had sufficient FCoV type I S glycoprotein incorporated in their envelope membrane to mediate transduction of primary feline, most probably monocyte, cells.

## DISCUSSION

The main conclusion of the experiments reported here is that fAPN does not function as a receptor for the attachment and entry of FCoV type I viruses. This is based upon the inability of retroviral pseudotypes bearing the FCoV type I S glycoprotein to transduce cells that express fAPN and can be transduced by retroviral pseudotypes bearing the FCoV type II S glycoprotein. This conclusion is consistent with the difficulties encountered in the isolation and propagation of type I FCoV in feline cell culture and is in agreement with the conclusions of Hohdatsu *et al.* (1992). However, it is not consistent with the report by Tresnan *et al.* (1996) that the UCD-1 strain of FIPV, a type I virus, is able to infect and express viral antigens in hamster and mouse cells that had been stably transfected with fAPN cDNA. It is, perhaps, relevant to note that in the experiments reported by Tresnan *et al.* (1996), the infection of fAPN-transfected hamster cells was much lower with type I viruses, compared with type II viruses (2 and 30 % of cells in tranfected cultures, respectively). At the moment, we can only suggest that the expression of fAPN in mouse or hamster cells, or the selection of cells in G418 is able to augment the infection of a small percentage of cells by a fAPN-independent route.

Also, in apparent contradiction to our results, there are several reports stating that type I FCoV can infect Fcwf-4 cells (Hohdatsu *et al.*, 1998; Jacobse-Geels & Horzinek, 1983), a cell line that we were unable to transduce with FCoV type I pseudotypes. It is noteworthy, however, that in these studies, the type I viruses used showed only a low level of cell-associated infection and that attachment or adsorption of virus to these cells could be significantly enhanced by low-speed centrifugation or the inclusion of polymeric cations, such as DEAE-dextran in the culture medium during the absorption period (Hohdatsu *et al.*, 1995). Our interpretation is that the FCoV type I S glycoprotein–receptor interaction at the surface of Fcwf-4 cells is of low affinity and is not sufficient to mediate a level of pseudotype transduction that is detectable in our system. Also, it is possible that in the absence of a high affinity receptor-mediated entry mechanism, the phagocytic activity of the macrophage-like Fcfw-4 cell line rapidly depletes the culture supernatant of retroviral pseudotypes.

The results of this study also suggest that the viral pseudotypes reported here may be useful for the identification of the cellular receptor for type I FCoV and the development of cell lines that would efficiently replicate and propagate type I FCoV. In the first instance, the viral pseudotypes could be used to screen for continuous feline cell lines that are permissive for attachment and entry. However, type I FCoV appears to have an extremely narrow host range and cell tropism, and we consider it probable that even if such a cell line was found, it would be unlikely to be useful for non-cell-associated virus replication. The most promising candidate would probably be feline gut epithelial cell line because FCoV is primarily an enteric virus that binds to the apical surface of gut epithelial cells in the feline gastrointestinal tract (Rossen *et al.*, 2001). Unfortunately, there are no feline gut cell lines available commercially. An alternative strategy would be to identify the FCoV type I virus receptor and to produce a permissive cell line that expresses this receptor at its cell surface. Again, the viral pseudotypes could be used to identify cells, for example in gut explant or peripheral blood monocyte cell cultures, that express the type I FCoV receptor and then to use these cells as a source of receptor protein-enriched membranes or receptor mRNA-enriched RNA. From this point onwards, the identification of the FCoV receptor, the production of a FCoV receptor cDNA and the generation of a stably transfected, receptor-expressing feline cell line could follow strategies that have proven successful for other viruses (Assavalapsakul *et al.*, 2006; Tatsuo *et al.*, 2000). Such a cell line would be a valuable tool for future FCoV studies.

## Supplementary Material

[Supplementary tables]
