# Supplementary material for: Type I feline coronavirus spike glycoprotein fails to recognize aminopeptidase N as a functional receptor on feline cell lines
Source: J Gen Virol. 2007 Jun;88(Pt 6):1753–60. doi: 10.1099/vir.0.82666-0 (PMC2584236; doi:10.1099/vir.0.82666-0)
Supplement: [Supplementary tables] [file supp_88_6_1753__index.html]

 Type I feline coronavirus spike glycoprotein fails to recognize aminopeptidase N as a functional receptor on feline cell lines -- Dye et al. 88 (6): 1753 Data Supplement - Supplementary tables -- Journal of General Virology

## 

### Type I feline coronavirus spike glycoprotein fails to recognize aminopeptidase N as a functional receptor on feline cell lines, by C. Dye, N. Temperton and S. G. Siddell

*Journal of General Virology* vol. **88**, part 6, pp. 1753 - 1760

**Supplementary Table S1.** Oligonucleotide primers used for cloning and sequenceing FCoV S protein genes

**Supplementary Table S2.** Plasmids used to transfect HEK 293T cells for the production of retrovirla pseudotypes   
  
[Single PDF file]  (24 KB)

  
  
