# Supplementary material for: Type I feline coronavirus spike glycoprotein fails to recognize aminopeptidase N as a functional receptor on feline cell lines
Source: J Gen Virol. 2007 Jun;88(Pt 6):1753–60. doi: 10.1099/vir.0.82666-0 (PMC2584236; doi:10.1099/vir.0.82666-0)
Supplement: [Supplementary tables] [file supp_88_6_1753__1.pdf]

**Supplementary Table S1.** Oligonucleotide primers used for cloning and sequencing FCoV S protein genes

| Primer | Use | Nucleotide sequence (5' to 3') | Position* |
|---|---|---|---|
| T003 | Forward primer for amplification of the truncated Type II S gene | GGCGGTACCGCCACCATGATTGTGCTCGTAACTTGCC | 20204-20227 on FIPV 79-1146 |
| T004 | Reverse primer for amplification of the truncated Type II S gene | GGCCTCGAGCTATTCAAATTGTCTTCTACTACATATAGAGTGAC | 24497-24528 on FIPV 79-1146 |
| T009 | Forward primer for amplification of the full-length Type II S gene | GGCGGTACCGCCACCATGATTGTGCTCGTAACTTGCCTCTTGT | 20204-20233 on FIPV 79-1146 |
| T010 | Reverse primer for amplification of the full-length Type II S gene | GGCCTCGAGTTAGTGGACATGCACTTTTTCAATTGGTTCA | 24534-24564 on FIPV 79-1146 |
| T013 | Forward primer for amplification of the truncated and full-length Type I S genes | GGCGGTACCGCCACCATGATCTTGTTACTAGCGCTTTTTAGTGTTGT | 20384-20419 on FCoV C1Je |
| T014 | Reverse primer for amplification of the truncated Type I S gene | GGCCTCGAGCTATTCAAATTGTCTTATACTGCAAAGAGAATGAC | 24724-24755 on FCoV C1Je |
| T015 | Reverse primer for amplification of the full-length Type I S gene | GGCCTCGAGTTAATGAATGTGAACCTTCTCAATGGG | 24765-24793 on FCoV C1Je |
| pCAGG-F | Sequencing the proximal plasmid-insert junction of the pCAGGS plasmid | ACGTGGTTGGTTAATTGTGCTGTC | 1662-1686 on pCAGGS |
| pCAGG-R | Sequencing the distal plasmid-insert junction of the pCAGGS plasmid | TCCCATATGTCCTTCCGAGTGA | 1886-1908 on pCAGGS |
| M13-F | Sequencing the proximal plasmid-insert junction across the TOPO cloning site | CAGGAAACAGCTATGAC | 433-448 on pCR-Blunt II-TOPO |
| M13-R | Sequencing the distal plasmid-insert junction across the TOPO cloning site | GTAAAACGACGGCCAG | 205-221 on pCR-Blunt II-TOPO |

* FIPV 79-1146, Acc. No. DQ010921; FCoV C1Je, Acc. No. DQ848678; pCAGGS, Riken Bioresources Center, pCR-Blunt-II-TOPO, Invitrogen

**Supplementary Table S2.** Plasmids used to transfect HEK 293T cells for the production of retroviral pseudotypes

| Pseudotype | Surface protein | Components | Constituent Plasmid DNA | DNA added to transfection |
|---|---|---|---|---|
| MLV(VSV-G) | VSV-G | Gag-Pol | cMVi | 400ng |
| | | GFP | cNCG | 1000ng |
| | | VSV-G | pMDG | 1000ng |
| MLV(bald) | none | Gag-Pol | CMVi | 400ng |
| | | GFP | cNCG | 1000ng |
| | | Empty | pCAGGS | 500ng |
| MLV(FCoVII-Str) | FCoV type II S (truncated) | Gag-Pol | cMVi | 400ng |
| | | GFP | cNCG | 1000ng |
| | | 79-1146 S | pCAGGS/FCoVSIItr | 500ng |
| MLV(FcoVI-Str) | FCoV type I S (truncated) | Gag-Pol | cMVi | 400ng |
| | | GFP | cNCG | 1000ng |
| | | C1Je S | pCAGGS/FCoVSItr | 500ng |

**Dye, C., Temperton, N. and Siddell, S. G. (2007).** Type I feline coronavirus spike glycoprotein fails to recognize aminopeptidase N as a functional receptor on feline cell lines. *J Gen Virol* **88**, 1753–1760.
